# Circular RNAs in renal cell carcinoma: from mechanistic to clinical perspective

**DOI:** 10.1186/s12935-023-03128-w

**Published:** 2023-11-22

**Authors:** Chunjie Huang, Pooya Esfani Sarafraz, Parisa Enayati, Elham Mortazavi Mamaghani, Emad Babakhanzadeh, Majid Nazari

**Affiliations:** 1 School of Medicine, Nantong University, Nantong, China; 2 Department of Pharmacy, Guilan University of Medical Sciences, Rasht, Iran; 3 Biological Sciences Department, Northern Illinois University, DeKalb, Illinois USA; 4 Department of Medicine, Iran University of Medical Sciences, Tehran, Iran; 5 Department of Medical Genetics, Shahid Beheshti University of Medical Sciences, Tehran, Iran; 6 Department of Medical Genetics, Shahid Sadoughi University of Medical Sciences, 64155-65117, Yazd, Iran

**Keywords:** CircRNA, Renal cell carcinoma, Biomarker, Targeted therapy

## Abstract

CircRNAs, a special type of noncoding RNAs characterized by their stable structure and unique abilities to form backsplicing loops, have recently attracted the interest of scientists. These RNAs are abundant throughout the body and play important roles such as microRNA sponges, templates for transcription, and regulation of protein translation and RNA-binding proteins. Renal cancer development is highly correlated with abnormal circRNA expression in vivo. CircRNAs are currently considered promising targets for novel therapeutic approaches as well as possible biomarkers for prognosis and diagnosis of various malignancies. Despite our growing understanding of circRNA, numerous questions remain unanswered. Here, we address the characteristics of circRNAs and their function, focusing in particular on their impact on drug resistance, metabolic processes, metastasis, cell growth, and programmed cell death in renal cancer. In addition, the application of circRNAs as prognostic and diagnostic biomarkers will be discussed.

## Introduction

In 2020, kidney cancer led to nearly 180,000 new deaths worldwide, making it one of the most common cancers [1]. In the United States, it is the eighth most prevalent cancer, accounting for 4.2% of all new cancer diagnoses [2]. Renal cell carcinoma (RCC) is the most common subtype and accounts for between 60 and 80 percent of all primary renal malignancies [3]. Advanced RCC is associated with high morbidity and mortality, but recent advances in treatment have significantly improved the prognosis for RCC over the past decade [4]. In particular, immune checkpoint inhibitors (ICIs) has emerged as an effective and critical new approach in the treatment of kidney cancer [5, 6]. However, the growing evidence of the efficacy of these treatments must be weighed against their potential toxicity and the risks of overtreatment, especially as ICIs are now being studied in non-metastatic settings and in combination with other agents [7]. Therefore, the discovery of new biomarkers for tumors is an important and current clinical goal, and circRNAs have the potential to be valuable indicators of disease [8, 9].

Long-stranded noncoding RNAs (lncRNA, > 200 nt) and tiny RNAs, such as microRNA (miRNA), are two types of noncoding RNAs commonly categorized by size. Noncoding RNAs (ncRNAs) form a group of RNA that are not translated. Nevertheless, they play a critical role in various health conditions, particularly cancer [9]. Eukaryotes contain large numbers of CircRNAs, a class of naturally occurring long noncoding RNAs. In viroids, covalently closed CircRNAs were first found in 1976 [10]. They were later discovered to be present in the cytoplasm of eukaryotic cells, to have cell- and organ-specific expression, and to possess significant biological activities [11, 12]. As sequencing technology improved, the significance of circRNAs became more apparent. These specific lncRNAs harbor a distinctive covalent single-stranded closed loop arrangement without a 3ʹ poly(A) tail or 5ʹ cap, making them more durable than mRNAs and resistant to RNase A degradation [13]. CircRNAs exhibit dual roles in RCC, functioning as both oncogenes and tumor suppressors. Their primary mechanism of action involves acting as molecular sponges for microRNAs. CircRNAs control gene transcription, alternative splicing, and protein translation, act as microRNA sponges, and cooperate with RNA-binding proteins [14].

This review addresses the effects of circRNA production on the growth and progression of RCC and provides a basic overview of its principles. The capability of circRNAs as predictive and diagnostic biomarkers is also addressed. Future cancer therapies could benefit from the important insights and knowledge gained from a deeper understanding of circRNAs.

## CircRNAs

Sanger began studying circRNAs in 1976 [15]. NcRNAs, previously considered “unique signals,” have received considerable attention as technology and knowledge have evolved. Because of their particular circular structure, circRNAs are more resilient to destruction by nucleic acid exonucleases than linear RNAs. The structure has remained largely unchanged throughout development and evolution.

### Biogenesis of CircRNAs

The formation of circRNAs requires specific genomic features. Firstly, circRNA exons and their adjacent introns are notably long, averaging three times the length of canonical linear RNAs. Secondly, these extended introns must possess inverted complementary sequence elements, such as inverted repeat Alu elements, which facilitate the close proximity of downstream 5ʹ-donor and upstream 3ʹ-acceptor splice sites [16]. It is worth noting that the majority of circRNAs are found within protein-coding genes and consist of complete exons, suggesting that their transcription is mediated by RNA polymerase II. The majority of circRNAs, specifically 84%, are derived from protein-coding genes. Among these circRNAs, 85% align in the same sense direction as exons of both coding and non-coding genes, while 10% align in the opposite antisense direction. The remaining 5% of circRNAs align to untranslated regions, introns, or gene loci that have not been annotated [17]. CircRNAs formed through back-splicing, a process in which the 3ʹ splice site of a downstream exon is joined to the 5ʹ splice site of an upstream exon, creating a continuous loop structure. This differs from linear RNAs, which have 5ʹ caps and 3ʹ polyadenylated tails [16].

**Fig. 1:**
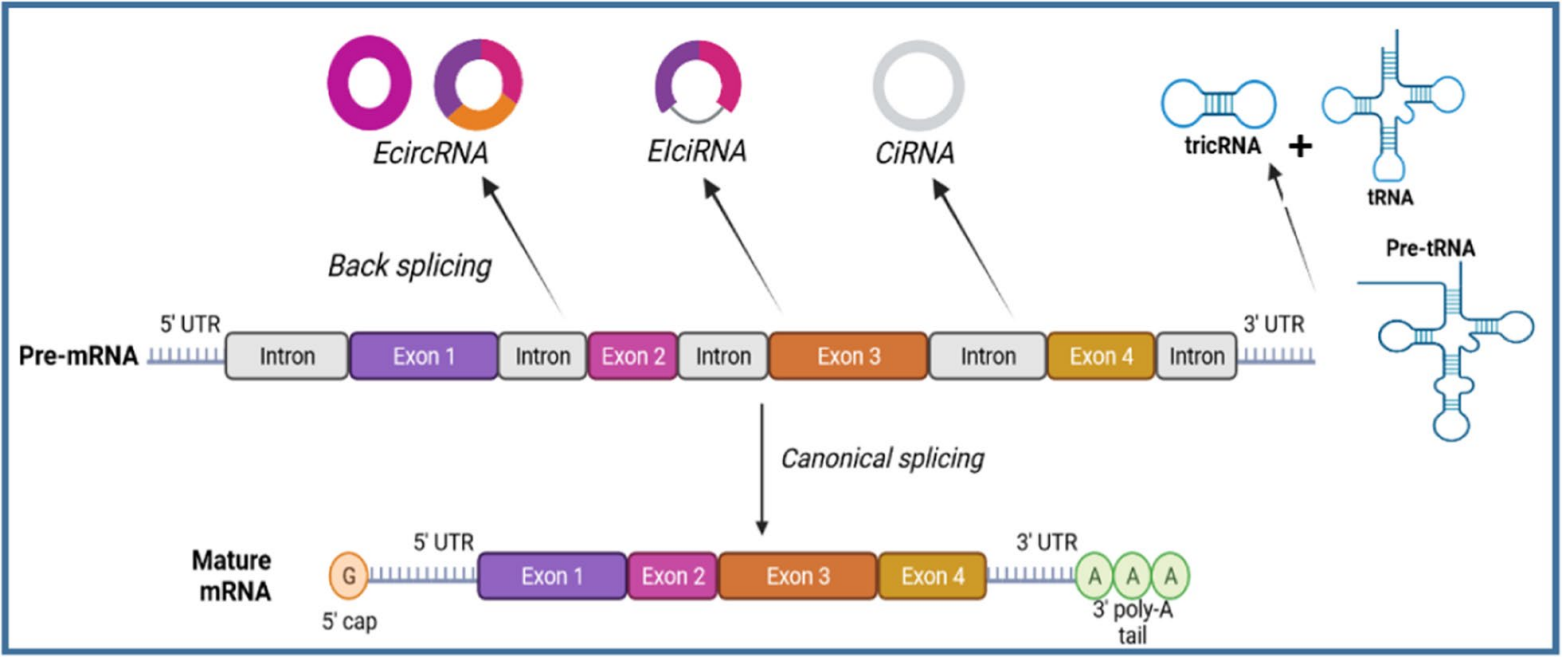
Biogenesis of CircRNA. Pre-mRNA experience either back-splicing, which produces both a circRNA and an alternatively spliced linear RNA without an exon, or typical splicing, which produces a linear RNA with an exon. CircRNAs can form in one of three ways during biogenesis: EIciRNAs, ecircRNAs, and ciRNAs. The process of direct backsplicing, in which the upstream 3ʹ splice acceptor site is covalently connected to the downstream 5ʹ splice donor site of the exon, leads to looping of the precursor mRNA, often resulting in ecircRNAs. Direct backsplicing is also used by EIcircRNAs, although backsplicing leaves certain intron sequences in the circRNA instead of deleting them. Removal of introns during splicing of pre-mRNA is thought to be the cause of ciRNA formation. TricRNA is a type of circular RNA formed by the splicing process of precursor tRNA

As a result of splicing of mRNA, precursor transfer RNAs (pre-tRNAs), intron circular RNAs (ciRNAs), exon circular RNAs (ecircRNAs), exon intron circular RNAs (eIcircRNAs), and tRNA intron circular RNAs (tricRNAs) are produced (Fig. 1). Most circRNAs are ecircRNAs, which are primarily placed in the cytoplasm. CiRNAs and EIcircRNAs, on the other hand, are mostly found in the nucleus. Standard models for circRNA loops include direct splicing back, lasso-driven circularization, and circularization mediated by RNA-binding proteins [14]. EcircRNAs are predominantly produced by direct backsplicing, a cleavage process in which a loop is formed in the precursor mRNA by covalently linking the upstream 3' splice acceptor site to the downstream 5ʹ splice donor site of the exon. This procedure creates a closed circRNA loop by generating a lariat splice loop, followed by shearing of the intron [18]. EIcircRNAs also apply the direct backsplicing method, but during backsplicing, some intron sequences are retained within the circRNA instead of being removed [19].

It is assumed that ciRNAs arise from the synthesis of lassos from introns deleted during splicing of the pre-mRNA. Synthesis of these ciRNAs can be amplified by expression vectors that rely on a consensus motif consisting of an 11 nucleotide C-rich component near the branch point and a 7 nucleotide GU-rich sequence near the 5ʹ splice site [20]. RNA-binding proteins (RBPs) contributed in circRNA formation include RNA-editing enzyme, muscle blinding protein 1, and fusion sarcoma protein [21, 22]. RBPs can dimerize and drive exon loops by binding to specific motifs in nearby introns [22]. Comprehensive transcriptome analyses revealed that 60% of human genes produce both linear and circular transcripts [23]. Canonical splice sites are used for circRNA biogenesis, and linear splicing of mRNAs competes with back-splicing. CircRNA transcription levels are often lower than mRNA transcription levels. Under normal physiological conditions, circRNA expression increases when backsplicing activity decreases and the efficiency of spliceosome components is reduced [24]. Exons in circular RNAs and the introns surrounding them are longer than those in linear RNAs. These elongated introns include inverted complementary sequence elements, for instance inverted Alu components, that promote the proximity of upstream 3-acceptor and downstream 5-donor splice sites [16].

### CircRNA degradation and exosome release

**Fig. 2:**
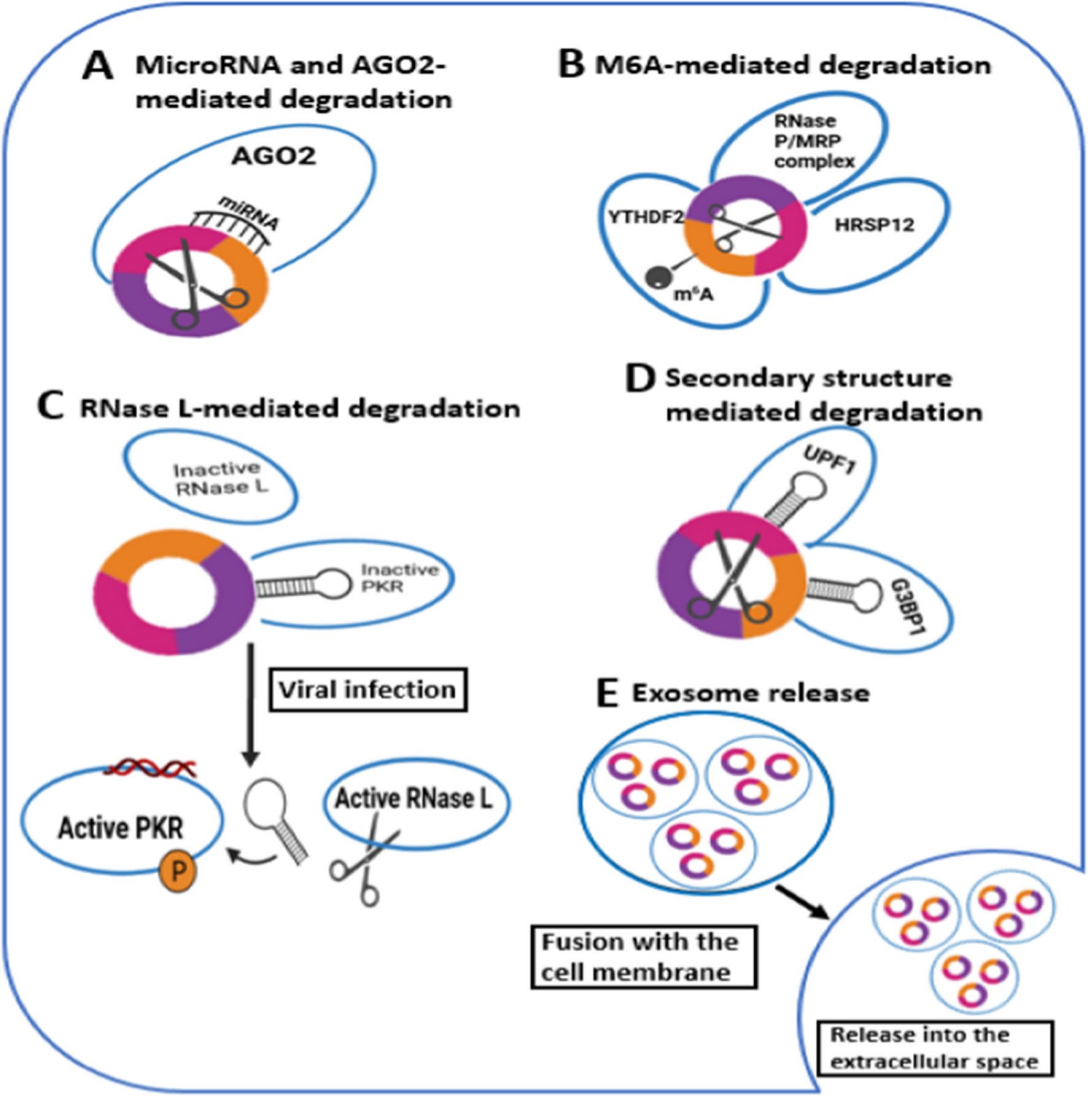
Disintegration of CircRNA and Extracellular Vesicle Discharge. **A** Certain circular RNAs (circRNAs) may be subject to degradation through targeted microRNA (miRNA) interaction, followed by argonaute-2 (AGO2)- facilitated RNA slicing. **B** CircRNAs carrying N6-methyladenosine (m6A) alterations may be detected and severed by the HRSP12-YTHDF2–RNase P/MRP complex. **C** RNA duplex structures (16–26 base pairs) of CircRNA may latch onto and hinder the functionality of double-stranded RNA-triggered protein kinase (PKR). In viral infection, RNase L is produced, breaking down the circRNAs, leading to PKR release and activation, crucial in the early stages of the innate immune response. **D** RNA binding proteins like Ras GTPase-activating protein-binding protein 1 (G3BP1) and regulator of nonsense transcripts 1 (UPF1) can bind via secondary structure mediation to unravel circRNAs, allowing UPF1's helicase activity to cleave them. **E** CircRNAs may be encapsulated in exosomes and expelled into the extracellular region following the fusion of multivesicular endosomes with the cellular membrane

Because of their unique circular structure, circRNAs are largely resistant to degradation. However, our understanding of the mechanisms by which they are degraded is still evolving. The mechanisms of circRNAs disintegration and extracellular vesicle discharge are summarized in Fig. 2. Certain circRNAs are degraded when they bind to miRNAs and are cleaved by Argonaute-2 (AGO2) [25]. Others are degraded by certain RNases after N6-methyladenosine (m6A) modification [26]. The core complex consisting of the N6 adenosine methyltransferase METTL3-METTL14 can make m6A modifications to circRNA transcripts, allowing cleavage by the YTHDF2-HRSP12-RNaseP/MRP complex in the cytoplasm [27].

Several decay mechanisms are linked to the secondary structures of circRNAs [28]. In cases where there is no viral infection, circRNA-RNA duplexes bind and hamper double-stranded RNA-activated protein kinase (PKR) due to ribonuclease L (RNase L) inactivity. RNase L cleaves circRNAs during viral infection, releasing and activating PKR, which is an important component of the initial innate immune response. Thus, activation of PKR is a direct consequence of circRNA degradation. Another degradation mechanism involves RBPs such as Ras GTPase-activating protein-binding protein 1 (G3BP1) and Regulator of Nonsense Transcripts 1 (UPF1) [29]. These RBPs bind circRNAs and unravel them, leading to their degradation, which is facilitated by the helicase activity of UPF1. This degradation depends on structure rather than RNA sequence.

When multivesicular endosomes fuse with the cell membrane, some circRNAs are packaged into tiny extracellular vesicles and released into the extracellular environment. [30–32]. It is possible that these circRNAs are also unleashed as extracellular vesicles, but this needs further investigation [33]. Exosomes usually contain more circRNAs than the cells from which they are derived. Because exosomes can be detected in the kidney and are released throughout the nephron, it is reasonable to conclude that changes in circRNA concentration in exosomes detected in urine could provide important information about overall renal health [34]. Therefore, circRNAs in urine could serve as valuable noninvasive biomarkers of kidney disease.

### Detection and identification of CircRNAs

Over 100,000 distinct circRNAs have been detected in human [35]. Their unique circular structure, lack of 3ʹ poly (A) tails and 5ʹ caps, and low abundance make detection by conventional methods difficult [36]. The first detections were made using Northern blotting and PCR methods [37]. Northern blotting, considered the gold standard, distinguishes circRNAs from linear RNAs by denaturing polyacrylamide gel electrophoresis and hybridization of RNA probes [16]. However, this technique suffers from low throughput and difficulties in detecting rare circRNAs [38]. RT -qPCR is commonly used for circRNA analysis but can produce misleading signals due to structural changes and rolling circle replication [39].

Newer techniques, such as rolling circle amplification (RCA), take advantage of the circular structure of circRNAs [40]. RCA offers simplicity, cost-effectiveness, and specificity in amplifying target circRNAs [41]. Enzyme-based detection reduces primer dependence by using duplex-specific nucleases (DSNs) to cleave DNA/circRNA hybrids, thereby enhancing fluorescence upon detection of the target circRNA [42]. NanoString Technologies’ nCounter assay allows accurate quantification of circRNA by hybridization with two probes. However, it requires expensive equipment [43]. Microarrays offer high-throughput detection but can pose problems when comparing data from different studies [44]. The subcellular localization of RNA can be determined by fluorescence in situ hybridization (FISH), which is, however, expensive and time-consuming [44, 45]. RNA-seq technology has greatly improved the identification of circRNAs [46]. However, circRNAs need to be biochemically enriched before short-read deep sequencing due to their low in vivo abundance and lack of poly (A) tails [47]. RiboRNA-seq provides valuable expression data but cannot differentiate signals from linear and circRNAs in exonic regions [48]. Small circRNAs can be detected using poly (A)-RNA-seq, but unusual circular isoforms cannot be precisely identified or quantified using this method [49]. RNase R-RNA-seq can improve circRNA enrichment compared with ribo-or poly (A)-RNA-seq [16]. Overall, an efficient, accurate, and sensitive method for circRNA research remains to be developed.

### Functions of CircRNAs

#### MiRNA sponges

**Fig. 3:**
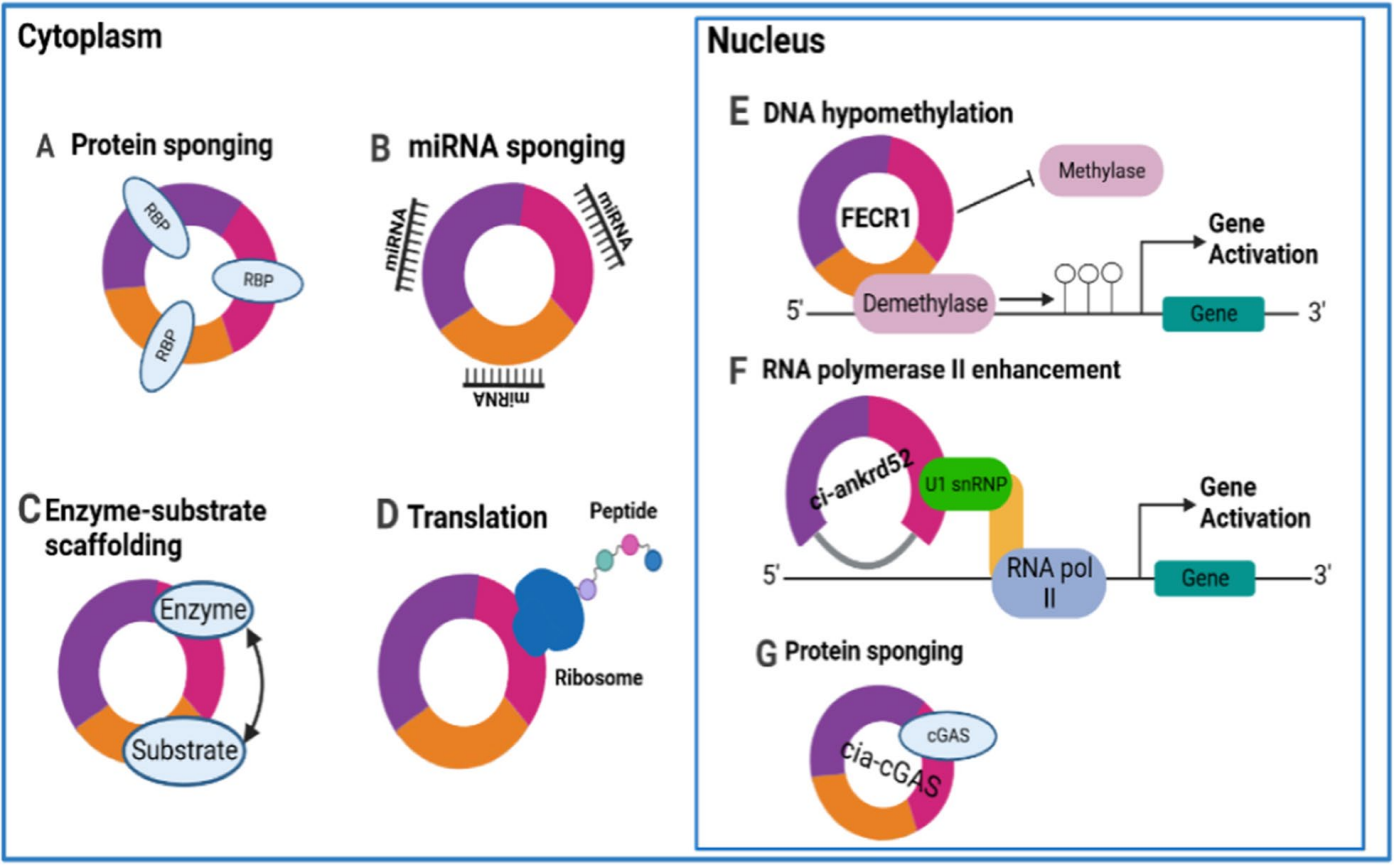
CircRNAs have multiple functions, such as: **A** they sponge the molecular function of RNA-binding proteins through specific interactions; **B** they act as miRNA sponges in the cytoplasm by binding miRNAs and preventing them from repressing target messenger RNAs; **C** By serving as a scaffold for interactions between enzymes and substrates, they improve reaction kinetics; **D** They are translated using rolling circle amplification when they have internal ribosome entry sites or m6A RNA modification of the 5ʹ-untranslated regions; **E** In the nucleus, FECR1 enhance the activity of methylases and demethylases by attaching to the gene promoter-chromatin complex; **F** promote RNA polymerase II activity by cooperating with the small nuclear ribonucleoprotein U1 or promoting RNA Pol II elongation machinery, as observed with ci-ankrd52, sircEIF3J, and circPAIP2; **G** In the nucleus, cia-cGAS sponge cyclic GMP–AMP synthase (cGAS)

The role of circRNAs as miRNA sponges has been a major focus in research. An overview of functions of circRNAs has been displayed in Fig. 3. By binding to the 3ʹ-untranslated regions (3ʹUTR) of mRNA, miRNAs, a large class of small noncoding RNAs, can control gene expression and translation [50]. According to studies, circRNAs have the ability to bind to miRNAs and compete with mRNA for these interactions by serving as miRNA sponges and subtly influencing gene expression [51, 52]. This process increases mRNA production and subsequent protein translation [53]. CiRS-7, a well-studied circRNA, harbors over 70 miR-7 binding sites and reportedly binds to miR-139-3p, thereby promoting RCC progression and metastasis via the PI3K/AKT pathway [54]. Similarly, circEYA3 increases c-Myc expression by sequestering miR-1294, which leads to increased CDC42 expression and promotes the invasion and migration of clear cell renal cell carcinoma (ccRCC) [55]. CircFAT1 increases the expression of YES -associated protein 1 via its miR-375 binding sites, and its silencing decreases cell proliferation, migration and invasion [56]. CircRNA biogenesis competes with splicing of pre-mRNA and affects its expression. It depends on conventional splice sites and spliceosome mechanisms.

Research shows that circRNAs can regulate gene expression in various stages of different malignancies. EIcircRNAs enhance transcription of parental genes through interactions with the polymerase II complex and small nuclear U1 ribonucleoproteins (U1 snRNPs) [57]. CircDONSON activates the NURF complex to start the synthesis of SOX4, which promotes the growth of gastric cancer [58]. CircRHOT1 expression accelerates hepatocellular carcinoma (HCC) growth and metastasis by initiating NR2F6 transcription. Suppression of CircRHOT1 decreases proliferation and metastasis of hepatoma cells [59].

#### Interaction with proteins

Besides their role as miRNA sponges and interaction partners of polymerase II complexes, circRNAs also possess the ability to bind proteins [60]. In their function as protein sponges, circRNAs can indirectly modulate RNA, with certain circRNAs that have minimal miRNA-binding sites playing a critical role in protein binding. Interestingly, however, the binding of RBP to circular RNA is less tight than to linear mRNA [61]. CircFoxo3, for example, which is exceedingly expressed in noncancerous cells, is involved in cell cycle regulation [62]. It binds to P21 and CDK2 to form a ternary complex that inhibits cell cycle progression. Moreover, increased circAmotl1 expression in newborn cardiac tissue may enhance AKT activity and promote cardiomyocyte survival. As a result of circAmotl1 binding to PDK1, AKT, and AKT1 is phosphorylated and transported to the nucleus [63].

#### Translation of circRNAs

While circRNAs function as noncoding RNAs and typically lack the 5ʹ cap, 3ʹ poly (A) tail, or clear open reading frame (ORF) required for translational regulation, recent studies have shown that they can indeed code for proteins [64]. CircRNAs can not only bind proteins but also function as miRNA sponges and interacting parts of polymerase II complexes. This process is facilitated by a specific sequence that allows the incorporation of an artificial internal ribosome entry site (IRES) upstream of the start codon, which immediately initiates translation through the ribosome [65]. In addition, the presence of N6-methyladenosine (m6A)-methylated adenosine residues that bind directly to eIF3 has been shown to facilitate circRNA translation [64]. CircZNF609, a distinct regulator of myogenic cell proliferation, is a typical example. It features an ORF that has the same start codon as its linear equivalent and a stop codon in the ORF that is a product of backsplicing at the termination point. Association with heavy polysomes converts CircZNF609 into a protein in a manner that is both splice-dependent and cap-independent, shedding light on the occurrence of protein-coding circRNAs in eukaryotes [66]. In addition, Zhang and his team made a breakthrough discovery: circSHPRH is able to synthesize a new functional protein, SHPRH-146A, which plays an important role as a tumor suppressor in glioblastoma [67].

### The role of circRNAs in RCC

**Fig. 4:**
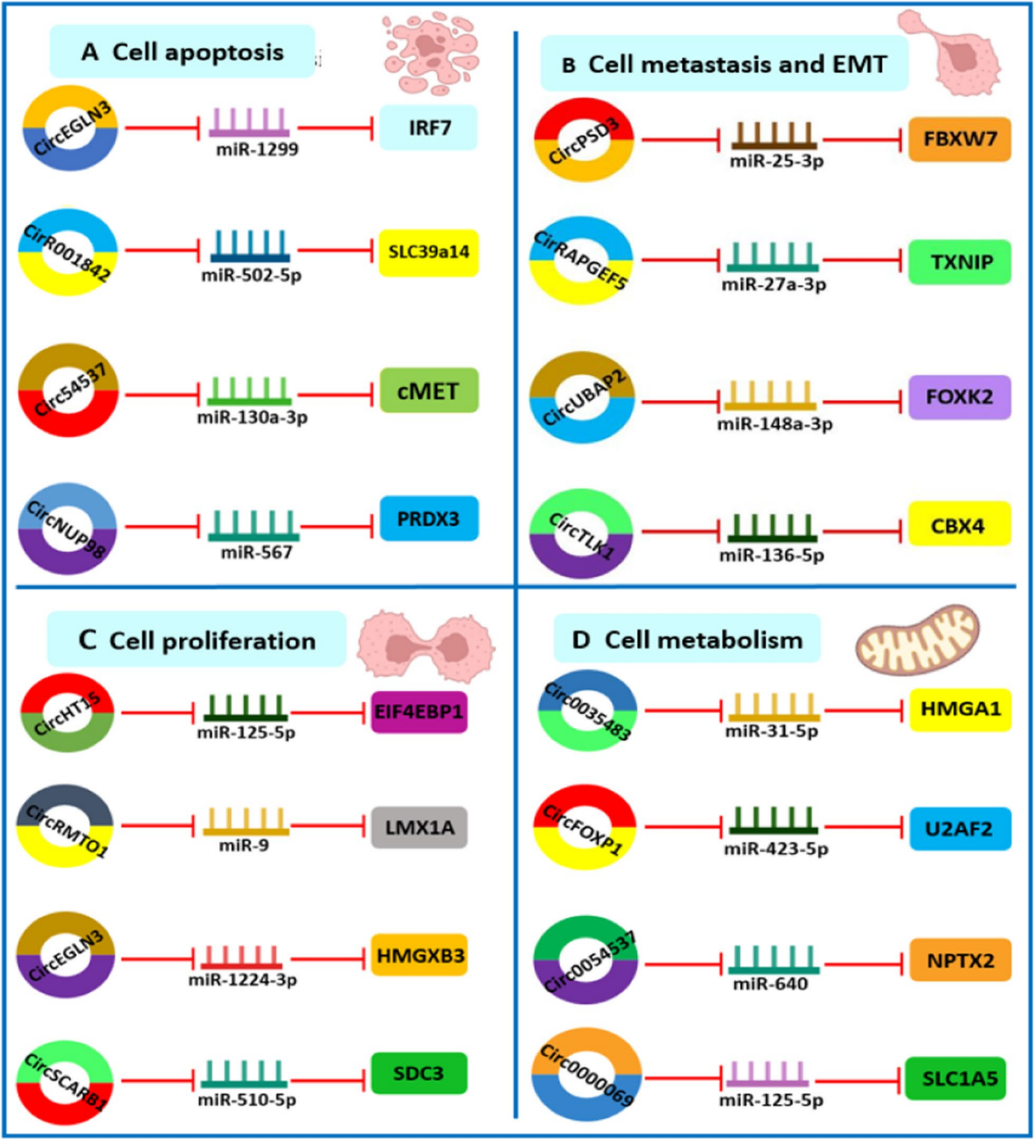
The role of CircRNAs in RCC through suppression of miRNAs and associated proteins. **A** Circ0054537, CircEGLN3, Circ001842 and CircNUP98, have an effect on apoptosis of renal cancer cells. **B** CircRNAs play a role in epithelial-mesenchymal transition (EMT) and metastasis of RCC including CircPSD3, CircUBAP2, CircRAPGEF5, and CircTLK1. **C** CircRNAs play a role in the cell cycle and growth of renal carcinoma cells; CircSCARB1, CircMTO1, CircEGLN3, CircCHT15 and CircMTO1 affect the proliferation of renal cancer cells. **D** CircRNAs affect the function of renal cancer cells. Circ0035483, Circ0000069, CircFOXP1 and Circ0054537 affect the metabolism of renal cancer cells

CircRNAs are critical for the growth and progression of renal cancer. Through RNA-seq research, scientists have discovered about 2000 circRNAs that are differentially expressed in RCC [68]. These circRNAs are involved in a range of cellular functions in renal cells, such as cell proliferation, metastasis, epithelial-mesenchymal transition (EMT), apoptosis, and metabolism (Fig. 4). Table 1 provides most interesting reports of circRNAs in RCC. In the following section, we review how circRNAs regulate these cellular functions and the molecular causes underlying RCC.

**Table 1:** Interesting reports of circRNAs in RCC

CircRNA	Target miRNA	miRNA target gene/sponge	Expression	Function	Reference
Circ0005875	miR-502-5p	ETS1	Up	Cell Cycle( +) Metastasis( +) Apoptosis( −)	[69]
CircESRP1	miR-3942	CTCF	Down	Cell Cycle(-) Metastasis(-)	[70]
CircCHST15	miR-125a-5p	EIF4EBP1	Up	Cell Cycle( +) Metastasis( +)	[71]
CircSDHC	miR-127-3p	CDKN3/E2F1	Up	Cell Cycle( +) Metastasis( +)	[72]
CircTLK1	miR-136-5p	CBX4	Up	Cell Cycle( +) Metastasis( +)	[73]
CircMTO1	miR-9	LMX1A	Down	Cell Cycle(-) Metastasis(-)	[74]
CircUBAP2	miR-148a-3p	FOXK2	Down	Cell Cycle(-) Metastasis(-)	[75]
CircSCARB1	miR-510-5p	SDC3	Up	Cell Cycle( +) Metastasis( +)	[76]
Circ0054537	miR- 130a-3p	cMET	Up	Cell Cycle( +) Metastasis( +)	[66]
Circ0001368	miR-492	LATS2	Down	Cell Cycle(-) Metastasis(-)	[77]
CircRAPGEF5	miR-27a-3p	TXNIP	Down	Cell Cycle(-) Metastasis(-)	[78]
CircEGLN3	miR-1224-3p	HMGXB3	Up	Cell Cycle( +) Metastasis( +)	[79]
Circ000926	miR-411	CDH2	Up	Cell Cycle( +) Metastasis( +)	[80]
CircAKT3	miR-296-3p	E-cadherin	Down	Metastasis(-)	[81]
Circ0035483	miR-31-5p	HMGA1	UP	Glycolysis( +)	[82]
CircFOXP1	miR-423-5p	U2AF2	UP	Glycolysis( +)	[83]
Circ0054537	miR-640	NPTX2	UP	Glycolysis( +)	[84]
Circ0000069	miR-125a-5p	SLC1A5	UP	Cell Cycle( +) Metastasis( +)	[85]

### Cell cycle

Research suggests that dysregulated circRNA expression is associated with uncontrolled proliferation of renal cancer cells and interruption of cell cycle control. For example, increased circ000926 expression in renal cancer cells correlates with unfavorable patient outcome [80]. Suppression of Circ 000926 hinders xenograft growth and reduces renal cancer cell growth. By directly inhibiting miR-411, it serves as a miRNA sponge and enhances CDH2 expression. When CircEGLN3, which is overexpressed in renal cancer, is suppressed, proliferation of ACHN and 769-P renal cancer cells is reduced [79]. CircEGNL3 functions as a miRNA sponge for miR-1224-3p, which consequently increases the expression of HMGXB3, decreases the inhibitory effect of miR-1224-3p on renal cancer, and promotes cell cycle and metastasis. In separate studies, circSCARB1 levels were shown to be increased in various RCC cell lines and RCC tissue [76]. CircSCARB1 interaction with miR-510-5p and SDC3 (a miR-510-5p target) facilitates cell growth inhibition caused by circSCARB1 silencing.

Simultaneous transfection with SDC3 partially counteracts the regulation of cell proliferation, whereas transfection with a miR-510-5p mimic decreases both SDC3 expression and cellular expansion. Moreover, overexpression of circMTO1 reduces the growth of 786-O and A498 renal cancer cells, whereas suppression of circMTO1 accelerates the development of OS-RC-2 and SN12C renal cancer cells [74]. MiR-223 and miR-9 are absorbed by circMTO1, which decreases their levels. Inhibition of circMTO1 promotes RCC cell growth by reducing LMX1A, a target of miR-9. MiR-9 has been shown to control LMX1A expression in RCC by transfection with a miR-9 mimic. Overexpression of a miR-9 inhibitor and LMX1A counteracts the tumour-promoting effect of circMTO1 silencing. Similarly, circTLK1 was discovered as a novel circRNA candidate formed by the TLK1 gene by high-throughput sequencing of circRNAs in renal cancer cell lines [73]. RCC cells have high expression of CircTLK1, and its silencing inhibits RCC cell growth. RCC patients with advanced TNM stage and high circSDHC expression in tissues detected by GSE100186 and GSE137836 sequencing have a poor prognosis [72]. In vivo and in vitro and, circSDHC promotes tumor cell proliferation.

Competition between circSDHC and miR-127-3p for binding was demonstrated using luciferase reporter assays and RNA pulldown to prevent suppression of E2F1 and CDKN3 signaling pathways leading to metastasis of RCC. When circSDHC expression is reduced, CDKN3 expression also decreases, suppressing E2F1 signaling, which can be restored by using a miR-127-3p inhibitor. CirCHT15 has been shown to be an accurate predictor of both overall survival (OS) and progression-free survival (PFS) after surgical resection of ccRCC [72]. Both renal cancer cell lines and ccRCC tissues show significant expression level of CirCHT15. Data from in vivo and in vitro experiments propose that CirCHT15 is a factor in the growth of ccRCC cells. CirCHT15 controls EIF4EBP1 expression through direct interaction with miR-125a-5p. CircDVL1 is not positively associated to the malignant characteristics of ccRCC and is expressed at low levels in sera and tissues of ccRCC cases [86]. Overexpression of circDVL1, which also leads to arrest of G1/S phase, prevents proliferation of many ccRCC cells. In ccRCC cells, circDVL1 acts as a miRNA sponge for oncogenic miR-412-3p and blocks its ability to inhibit PCDH7.

### Metastasis and EMT

Cancer cell invasion and metastasis, two crucial processes in the development and progression of renal cancer, have been found to be regulated by circRNAs [87]. Thus, a possible link between reduced circPSD3 levels and tumor metastasis in ccRCC patients is suggested by a decrease in circPSD3 expression in ccRCC tissues [88]. CircPSD3 significantly reduces cell invasion, migration and EMT under laboratory conditions and prevents lung metastasis in animal models. CircPSD3 and miRNA-25-3p interact in regulating FBXW7 expression. CircPSD3 serves as a potential target for the diagnosis and treatment of ccRCC by blocking the miR-25-3p/FBXW7 pathway, thereby arresting tumor growth. Despite uncertainty about circRAPGEF5 role in RCC, a study with data from 245 patients with this disease found a favorable correlation between aggressive clinical features and decreased circRAPGEF5 expression [78]. The circRAPGEF5/miR-27a-3p/TXNIP pathway was observed to inhibit RCC growth and progression. In ACHN, 786-O, and A498 cell lines and RCC tissues, one study discovered that circ0001368 expression was reduced [77].

The findings indicate that circ0001368 targets miR-492 which directly effects LATS2 by binding to the 3ʹUTR region of LATS2. Overexpression of miR-492 promoted cell invasion, while suppression of miR-492 inhibited it. Prior studies have shown that LATS2 attenuates hepatocellular carcinoma via the DNMT1/EZH2 pathway and reduces metastasis of glioma cell by enhancing tafazzin [77]. Overexpression of LATS2 dramatically reduces the ability of ACHN and 786-O cells to proliferate and invade. By scavenging miR-492, circ0001368 increases LATS2 expression while reducing growth and invasion of renal cancer cells. Furthermore, the circSCARB1/miR-510-5p/SDC3 signaling pathway facilitates renal carcinoma cell invasion and migration [76]. In vivo and in vitro studies have shown that circUBAP2 expression is significantly reduced in ccRCC [75]. Significant reduction in ccRCC cell invasion and migration is achieved by increased circUBAP2 expression. MiR-148a-3p counteracts the inhibitory effect of CircUBAP2 on ccRCC cell invasion and migration. CircUBAP2 serves as miRNA sponge for miR-148a-3p. Moreover, the miR-148a-3p target gene FOXK2 has been shown to suppress ccRCC cells by reversing the effect of miR-148a-3p inhibitor [75]. Moreover, circUBAP2 controls the miR-148a-3p/FOXK2 pathway to significantly limit tumor growth in ccRCC cells [75]. In ccRCC, circAKT3 was consistently downregulated. Sequencing of 60 ccRCC tissues, surrounding normal tissues, ccRCC cell lines, and another 60 tissue samples led to this discovery [81]. Overexpression of circAKT3 prevented ccRCC metastasis, whereas knockdown of circAKT3 stimulated ccRCC invasion and migration. The miR-296-3p/E-cadherin signaling pathway is blocked by circAKT3, which prevents the spread of ccRCC.

According to one study, circTLK1 is mainly present in the cytoplasm, and high expression positively correlates with distant metastases and poor prognosis for patients. MiR-136-5p sponging positively controls expression of CBX4 [73]. The suppression of RCC cell phenotype by prevention of circTLK1 is reversed by increased CBX4 expression. Moreover, there is a positive association between the expression level of VEGFA and CBX4 in RCC tissues. Significantly less VEGFA was expressed in RCC cells when CBX4 was knocked down. In another study, circESRP1 expression was shown to be low in renal cancer cells and tissues and was negatively related with tumor size, TNM stage, and distant metastasis of renal cancer [70]. CircESRP1 interacts with miR-3942 in a competitive manner that controls CTCF downstream. CTCF specifically enhances transcription of circESRP1, modulates the circESRP1/miR-3942 axis, and forms a positive feedback loop. By c-Myc-driven EMT, this signaling system controls the behavior of ccRCC cells. CircMYLK, which is particularly upregulated in RCC, associates with miR-513a-5p and promotes VEGFC production, facilitating tumorigenesis of RCC cells [89]. Metastasis of RCC is stopped by silencing circMYLK both under laboratory conditions and in animal models. Upregulation of circTXNDC11 is associated with advanced TNM stage and lymph node metastasis in RCC. Inhibition of circTXNDC11 slows cell invasion and metastasis under laboratory conditions [90]. By stimulating the MAPK/ERK pathway, CircTXNDC11 promotes RCC invasion and migration.

### Apoptosis

In renal cancer cells, circRNAs were discovered to have an effect on apoptosis. Increase in miR-130a-3p expression promotes RCC cell death because it is abnormally low in RCC cells [91]. Luciferase reporter assays and RNA pulldown proved that miR-130a-3p and circ0054537 directly interact. Circ0054537 and miR-130a-3p work together to control the oncogene c-Met, which is suppressed by miR-130a-3p and affects the development and spread of RCC tumors. Similarly, significant circSCARB1 expression was found in RCC cells, and knockdown of circSCARB1 causes apoptosis in A498 and 786-O cell lines [91]. Increased expression of Circ0005875 was observed in RCC. When this circular RNA is knocked down, the expression of miR-502-5p increases, leading to higher apoptosis propensity in renal cancer cells [69]. Sequencing revealed high circEGLN3 levels in RCC tissues and cell lines, indicating a negative prognosis for RCC patients [92]. RCC cells undergo apoptosis when CircEGLN3 is silenced. CircEGLN3, which is mainly placed in the cytoplasm, blocks RCC development by targeting miR-1299, which alters IRF7 level. Circ001842 is also overexpressed in RCC and contributes to disease development via an SLC39A14-dependent miR-502-5p pathway [93]. When circ001842 is silenced, RCC cells are driven into apoptosis. In another study, circNUP98 was found to be preferentially increased in 78 paired RCC tumors compared to nearby normal tissues. Inhibition of circNUP98 increased miR-567 expression, slowed RCC cell growth, and decreased PRDX3 levels [94]. In addition, STAT3 was found to promote circNUP98 in RCC cells. The new STAT3/circNUP98/miR-567/PRDX3 pathway exploits the CircNUP98 oncogene as a therapeutic target and possible biomarker for the treatment of RCC.

### Metabolism

CircRNAs have been displayed to control the metabolism of renal carcinoma cells. Circ0035483, for example, is overexpressed in renal cancer cells and tissues. Circ0035483 overexpression can promote glycolytic metabolism in RCC cells, as shown by the fact that a decrease in Circ0035483 expression reduces lactate production and glucose consumption in RCC cells [94]. In RCC cells, circ0035483 is turned off when miR-31-5p is overexpressed. By negatively regulating HMGA1, miR-31-5p inhibits the ability of RCC cells to become malignant. Due to the high expression of circFOXP1 in RCC tissues and cell lines, downregulation of circFOXP1 inhibits glycolysis in renal cancer cells [83]. The miR-423-5p/U2AF2 pathway is activated by ZNF263, which is upstream of circFOXP1 and promotes renal carcinoma development. According to this study, RCC tissues and cells had higher levels of circ0054537, which was mainly found in the cytoplasm [84]. Glycolysis is inhibited and apoptosis is promoted in RCC cells when the circ0054537 gene is knocked down. Circ0054537 attaches to miR-640 and targets NPTX2, as demonstrated by luciferase reporter.

Yang and colleagues reported that the level of circ0000069 was abnormally high in RCC tissues and cells. Reduction of circ0000069 led to a decrease in proliferation, metastasis, and glutamine metabolism of RCC cells [85]. In addition, circ0000069 was found to act as a sponge for miR-125a-5p, and inhibition of miR-125a-5p mitigated the impacts of circ0000069 reduction on the malignant behavior of RCC cells. The study also identified SLC1A5 as a target gene of miR-125a-5p. Higher expression of miR-125a-5p suppressed RCC cell progression, whereas increasing SLC1A5 counteracted this effect.

### Clinical application of circRNAs

Numerous studies have recognized the diagnostic capability clinical relevance of circRNAs expression in RCC. For instance, circ0001451 was highlighted for its promising diagnostic marker for ccRCC with an area under the receiver operating characteristic curve (AUC-ROC) of 0.70, complemented by a specificity of 0.60 and sensitivity of 0.75 [95]. CircHIPK3 was also identified as a potential diagnostic marker and has a remarkable AUC of 0.95 for ccRCC [96]. In addition, research suggests that the combination of circRNAs with linear transcripts may provide greater diagnostic value compared to single circRNAs. For example, Franz et al. identified circNOX4, circEGLN3, and circRHOBTB3 as potential diagnostic biomarkers. The AUC-ROC values of circRHOBTB3 and circNOX4 and in RCC tissues were 0.82 and 0.81, respectively [97]. However, circEGLN3 displayed higher reliability with an AUC-ROC of 0.98. More impressively, simultaneous detection of linEGLN3 and circEGLN3 resulted in an improved AUC-ROC of 0.99, corresponding to a sensitivity of 95% and a specificity of 99%. A meta-analysis by Rashedi et al. evaluated 8 studies with 604 RCC cases and 527 controls to investigate the diagnostic value of circRNAs for RCC detection. The results showed that circRNAs have the potential to be used as diagnostic biomarkers for RCC tissue samples, with a pooled sensitivity, specificity, and AUC of 0.84, 0.84, and 0.91, respectively. This suggests that circRNAs are highly accurate in detecting RCC in tissue samples. However, the accuracy of circRNAs as diagnostic biomarkers for body fluid (serum and urine) specimens was moderate, with a sensitivity, specificity, and AUC of 0.78, 0.69, and 0.71, respectively. This suggests that circRNAs are less accurate in detecting RCC in body fluid samples [98].

Certain circRNAs have been shown to be important prognostic biomarkers, influencing outcomes such as OS, DFS, and PFS. Li et al. reported that increased circPRRC2A expression was a distinct risk factor for decreased OS and metastasis-free survival [99]. It was also found that patients with RCC who have increased expression of circTLK1 [73], circNUP98 [94], and circ0085576 [100] have reduced OS and DFS rates. On the other hand, lower expression of circRAPGEF5 has been correlated with unfavorable OS and RFS in patients with RCC [78]. In addition, Frey et al. found that low circNETO2 and high circEHD2 levels independently predicted a decline in PFS, OS, and cancer-specific survival in patients with ccRCC undertaking nephrectomy [101]. In addition, it was observed that patients with ccRCC who had higher circ101341 and circ-ABCB10 expression showed worse OS than patients with lower expressions [102]. Similarly, Zeng et al. reported lower survival rates in patients with high circ001842 expression, suggesting a positive correlation with RCC severity [93]. Interestingly, circEHD2 is highly expressed in ccRCC tissues but rarely detected in normal adjacent kidney tissues that it may be a specific biomarker for prognosis of ccRCC patients. High circEHD2 levels are an independent prognostic factor for OS and PFS in patients with ccRCC [103]. A recent meta-analysis found that tumor promoter circRNAs were associated with reduced OS, while tumor suppressor circRNAs were linked with better OS in RCC patients. These results were consistent in the multivariate analysis. Similar findings were obtained for DFS/PFS/RFS, with tumor promoter circRNAs being correlated with poor DFS/PFS/RFS and tumor suppressor circRNAs being associated with improved DFS/PFS/RFS in the univariate analysis. These findings were also consistent in the multivariate analysis [98].

In a microarray study of ccRCC tissue, a circRNA signature involving circNOX4, circRHOBTB3, and circEGLN3 was identified as a potential prognostic biomarker for cancer-specific, recurrence-free, and overall survival, with a reference of [104]. This signature demonstrated improved predictive accuracy compared to clinical models based on clinicopathological factors. Notably, the expression of circEGLN3 showed a high accuracy of 97% in discriminating between malignant and normal tissue. Another study reported that increased expression of circEGLN3 was associated with shorter survival in patients with RCC, with a reference of [39]. In patients with ccRCC, the circRNA circ-ABCB10 was found to be upregulated in tumor tissues compared to adjacent tissues. High expression of circ-ABCB10 correlated with advanced pathological grade and TNM stage and served as an independent predictor of worse overall survival in these patients, with a reference of [105]. Furthermore, mRNA expression of METTL14 was found to negatively correlate with TNM stage and positively correlate with overall survival in patients with RCC, with a reference of [106]. Rashedi et al. analyzed 26 studies with a total of 2048 patients with RCC and found that 6 circRNAs were downregulated and 18 circRNAs were upregulated in RCC tissues. The downregulated circRNAs were associated with smaller tumor size, lower T stage, less lymph node metastasis, less distant metastasis, and lower TNM stage. The upregulated circRNAs were correlated with higher T stage, more lymph node metastasis, more distant metastasis, and higher TNM stage [98].

The problem of drug resistance is currently a major challenge in cancer therapy and requires urgent attention [107]. Circsnx6, a particular circular RNA, is particularly abundantly expressed in sunitinib-resistant RCC cells [108]. It is thought to play a role in sunitinib resistance by modulating the level of lysophosphatidic acid within cells. Since CircSNX6 interacts with the U1 snRNP to upregulate ME1 expression and induce drug resistance in RCCs, CircME1 could be used as a biomarker to predict sunitinib resistance and as a therapeutic target for ccRCC [109]. Gemcitabine resistance in RCC and the high expression level of circ0035483 are associated [110]. Gemcitabine, a chemotherapeutic agent that is a deoxycytidine nucleoside analog, shows remarkable therapeutic efficacy against RCC. However, drug resistance is often a major problem. Overexpression of circ0035483 has been found to stimulate gemcitabine resistance by increasing cyclin B1 expression by sequestering miR-335 in RCC [110]. In addition, reduced expression of circ0035483 was found to inhibit cellular glycolytic activity [82]. Increased glycolytic activity is a common feature of proliferating cells and is often targeted in renal cancer therapies. Therefore, circ0035483 has the potential to circumvent gemcitabine resistance and affect glycolytic activity in RCC treatment. Nevertheless, our knowledge of the role and mechanisms of circRNAs in the development of anticancer drug resistance is still at an early stage and remains to be fully explored.

One potential therapeutic approach involves modulating native pathogenic circRNAs through methods such as silencing or overexpression by artificial circRNAs. These artificial circRNAs can be engineered to act as miRNA sponges, generate circular versions of native linear RNAs with therapeutic effects, translate proteins, modulate the immune system, control protein activity (as aptamers), control transcription or splicing, and replicate autonomously following in vivo delivery [111]. Silencing circRNAs, such as apoptosis-related circRNA (circHIPK3), circFoxo3, and cZNF609, mitochondrialfission, and using short hairpin RNA has shown beneficial effects in various disease models [111]. The CRISPR-Cas genome editing system has also been used to generate a mouse model with knockout of ciRs-7 [112]. Other approaches to silencing circRNAs include using antisense oligonucleotides (AONs) that bind to circRNAs and inhibit their interactions with target molecules, as well as antagonists that block the molecular interactions of circRNAs by shielding binding sites for proteins or miRNAs [113]. On the other hand, overexpression of circRNAs can be achieved by packaging them into extracellular vesicles for use as delivery vectors or by injecting an expression plasmid conjugated with colloidal gold nanoparticles, as demonstrated for circFoxo3 [114].

Nanoparticles have emerged as potential delivery systems for circRNA-based therapeutics, offering advantages such as improved stability, enhanced intracellular entry, and reduced immunogenicity of these molecules. In a mouse study, gold nanoparticles (PEG-AuNPs) conjugated with short interfering RNA (siRNA) targeting circDnmt1 demonstrated the ability to effectively suppress tumor growth and cellular autophagy [115]. Another study revealed that using AuNPs to deliver AONs, which block the binding sites on circCcnb1 for Ccnb1 and Cdk1, resulted in inhibited tumor growth and increased mouse survival [116]. These findings highlight the potential of nanoparticles as promising delivery systems for circRNA-targeting agents in renal cancer. In addition to nanoparticles, exosomes have also been investigated as carriers for circRNA-based therapeutics and circRNA expression vectors. Exosomes, small vesicles secreted by cells, contain various biomolecules including circRNAs. They play a crucial role in intercellular communication and can be engineered to deliver therapeutic molecules to specific target cells. However, further research is necessary to optimize the properties of nanoparticles and exosomes to ensure efficient and effective delivery of circRNA-based therapeutics in the context of renal cancer [24].

Therapeutic approaches utilizing circRNAs have primarily been investigated in preclinical studies. However, several challenges must be addressed before fully realizing the therapeutic potential of these approaches [117]. One significant concern with RNA interference (RNAi)-based strategies is the potential for off-target gene silencing, similar to the miRNA-like effect induced by small molecules like siRNA. The targeting of transcripts by siRNA relies on partial complementarity, typically occurring between the 3ʹ UTR of the transcript and the seed region of the siRNA [118]. While circRNA knockdown experiments often confirm that corresponding linear mRNA levels remain unaffected, off-target effects beyond their linear counterparts are less predictable. Ongoing research focuses on designing siRNA molecules to mitigate off-target effects in RNAi approaches [119]. It is important to note that while many circRNAs exhibit tissue- or cell-specific expression patterns, some circRNAs are present in multiple tissue or cell types, posing challenges when employing common targeting strategies that may inadvertently affect off-target tissues or cells [120]. Another consideration is the safety of using PEG as vehicles for delivering circRNA-targeting agents or circRNA plasmids in animal models, as their potential clinical use remains uncertain due to inconsistent conclusions regarding their toxicity from previous studies [121]. While there is significant interest in applying circRNA research findings to clinical scenarios for early disease diagnosis and prognosis prediction, it is essential to conduct large-scale clinical studies to validate the reliability and sensitivity of these circRNA biomarkers.

## Conclusions

To thoroughly evaluate the therapeutic potential of circRNAs in RCC, further research is needed, including multicenter validation and larger sample size. The discovery of additional circRNAs and the elucidation of their biological functions and mechanisms are critical for the development of efficient therapeutic approaches. Challenges in circRNA therapy include improving vectors, cyclization techniques, delivery methods, chemical synthesis, and control methods. Advanced technologies such as CRISPR-Cas-based editing and single-cell RNA-Seq will further our understanding of circRNAs and their clinical utility. Despite these gaps in understanding, circRNAs are a promising tool for clinical diagnosis and treatment. This article reviews the biosynthesis, regulation, identification techniques, and role of circRNAs in renal cancer, including their effects on drug resistance, metabolism, apoptosis, tumor growth, and metastasis. Recent advancements have identified critical circRNAs in urologic malignancies, making circRNA-based tumor diagnosis and treatment attractive.

## Data Availability

Not applicable.
