# Correction: circular RNAs in renal cell carcinoma: from mechanistic to clinical perspective

**DOI:** 10.1186/s12935-023-03184-2

**Published:** 2024-01-18

**Authors:** Chunjie Huang, Pooya Esfandi Sarafraz, Parisa Enayati, Elham Mortazavi Mamaghani, Emad Babakhanzadeh, Majid Nazari

**Affiliations:** 1 School of Medicine, Nantong University, Nantong, China; 2 Department of Pharmacy, Guilan University of Medical Sciences, Rasht, Iran; 3 Biological Sciences Department, Northern Illinois University, DeKalb, IL USA; 4 Department of Medicine, Iran University of Medical Sciences, Tehran, Iran; 5 Department of Medical Genetics, Shahid Beheshti University of Medical Sciences, Tehran, Iran; 6 Department of Medical Genetics, Shahid Sadoughi University of Medical Sciences, Yazd, 64155-65117 Iran

**Correction to: Cancer Cell International (2023) 23:288**

10.1186/s12935-023-03128-w

In this article [1], the author’s name Pooya Esfandi Sarafraz was incorrectly written as Pooya Esfani Sarafraz.
